# Triethyl Citrate (TEC) as a Dispersing Aid in Polylactic Acid/Chitin Nanocomposites Prepared via Liquid-Assisted Extrusion

**DOI:** 10.3390/polym9090406

**Published:** 2017-08-31

**Authors:** Natalia Herrera, Anshu Anjali Singh, Asier M. Salaberria, Jalel Labidi, Aji P. Mathew, Kristiina Oksman

**Affiliations:** 1 Division of Materials Science, Composite Center Sweden, Luleå University of Technology, Luleå SE-97187, Sweden; natalia.herrera.vargas@ltu.se (N.H.); anshu.anjali.singh@ltu.se (A.A.S.); 2 Biorefinery Processes Research Group, Department of Chemical and Environmental Engineering, Faculty of Engineering, Guipúzcoa, University of the Basque Country, Plaza Europa 1, Donostia-San Sebastian 20018, Spain; asier.martinez@ehu.eus (A.M.S.); jalel.labidi@ehu.eus (J.L.); 3 Division of Materials and Environmental Chemistry, Stockholm University, Stockholm SE-10691, Sweden; aji.mathew@mmk.su.se; 4 Fibre and Particle Engineering, University of Oulu, Oulu FIN-90014, Finland

**Keywords:** chitin nanocrystals, biopolymer, nanocomposite, extrusion, dispersion

## Abstract

The production of fully bio-based and biodegradable nanocomposites has gained attention during recent years due to environmental reasons; however, the production of these nanocomposites on the large-scale is challenging. Polylactic acid/chitin nanocrystal (PLA/ChNC) nanocomposites with triethyl citrate (TEC) at varied concentrations (2.5, 5.0, and 7.5 wt %) were prepared using liquid-assisted extrusion. The goal was to find the minimum amount of the TEC plasticizer needed to enhance the ChNC dispersion. The microscopy study showed that the dispersion and distribution of the ChNC into PLA improved with the increasing TEC content. Hence, the nanocomposite with the highest plasticizer content (7.5 wt %) showed the highest optical transparency and improved thermal and mechanical properties compared with its counterpart without the ChNC. Gel permeation chromatography confirmed that the water and ethanol used during the extrusion did not degrade PLA. Further, Fourier transform infrared spectroscopy showed improved interaction between PLA and ChNC through hydrogen bonding when TEC was added. All results confirmed that the plasticizer plays an important role as a dispersing aid in the processing of PLA/ChNC nanocomposites.

## 1. Introduction

Polylactic acid (PLA) is an attractive biopolymer for packaging and biomedical applications because of its biodegradability, non-toxicity, good mechanical properties, high optical transparency, and its commercial availability. However, PLA is brittle, and it exhibits low thermal stability, low melt strength, moderate barrier properties, and a slow crystallization rate. It is, therefore, necessary to modify the PLA to improve these properties to make PLA competitive among the common polymers used in industry [1,2,3]. PLA has been mixed with plasticizers [4], polymers [5], layered silicates [6], carbonaceous nanomaterials [7], cellulose [8], chitin [9], or a combination of these materials resulting in hybrid composites [10].

The development of nanocomposites based on PLA and chitin can be a good approach to improve the properties of PLA and to produce fully bio-based and biodegradable materials. Chitin nanofibers and nanocrystals have been recently used as additives to enhance thermal and mechanical properties of polymers, such as chitosan [11], natural rubber [12], poly(ethylene oxide) [13], thermoplastic starch [14], polypropylene [15], poly(caprolactone) [16], and poly (lactic acid) [17,18]. However, some problems and challenges still remain for the development of these materials into a large-scale process and, thus, for commercial use. 

Generally, the most common method to prepare chitin nanocomposites has been solvent casting [11,12,13], but melt-compounding is also used [9,16,17,18]. Melt compounding is an interesting processing technique if industrial applications are targeted because it can be scaled up. However, the extrusion of nanocomposites is challenging because of the tedious feeding of dried nano-sized materials into the extruder and their high tendency to aggregate when dried because of their high surface area, and in the case of chitin, also due to the formation of hydrogen bonds. In addition, poor compatibility between the hydrophobic matrices, such as PLA, and the hydrophilic additives, such as chitin, is challenging. All of these aspects hinder the dispersion and distribution of nanomaterials into the matrix, affecting the final properties of nanocomposites. 

Surface modifications of chitin by coupling agents [19], acylation [20], oxidation [21], and polymer grafting [22] are some of the approaches that are used to improve the interfacial adhesion between chitin and polymers. However, surface modifications are expensive and time consuming and do not solve all the above mentioned problems. 

We are proposing a simpler approach to solve the problems with processing, handling of dried nanomaterials, as well as poor compatibility between different phases. This approach entails feeding the nanomaterial as a liquid together with a plasticizer and solvents into the extruder. It is expected that the plasticizer covers the nanomaterial and, thus, prevents its aggregation during the process when the solvents evaporate. We have demonstrated in our previous studies that chitin nanomaterials prepared via the liquid-assisted extrusion resulted in well-dispersed and distributed nanomaterials in the PLA matrix [9,17].

The use of plasticizers in PLA nanocomposites has been studied by several researchers [8,18,23,24,25,26,27]. For example, Oksman et al. [23] reported that the dispersion of cellulose nanocrystals was enhanced when poly(ethylene glycol) (PEG) was used as a processing aid. Qu et al. [25] reported that cellulose nanofibers where more evenly dispersed in the PLA when PEG was used. Similarly, Herrera et al. [8] showed that the addition of cellulose nanofibers together with a glycerol triacetate plasticizer resulted in PLA with improved toughness due to well-dispersed nanofibers. Wang et al. [24] showed that the size of carbon black agglomerations in the polylactic acid matrix was decreased when acetyl tributyl citrate (ATBC) was added. Chieng et al. [27] reported enhanced toughness for the PLA when graphene nanoplatelets were incorporated with PEG. Recently, Li et al. [18] described that PEG, as well as polyethylene oxide (PEO), were used as a pretreatment for chitin nanofibers and indicated that both additives improved the interfacial adhesion between the nanofibers and PLA. Erpek and Yilmazer [26] used PEG as a compatibilizer for halloysite nanotubes (HNT) in PLA. However, unlike the other studies, in this case, PEG did not contribute to the dispersion of HNT. These above-mentioned studies showed that the use of plasticizer enhances the dispersion and distribution of nanomaterials, such as cellulose, chitin and carbon black, into PLA. However, it is well known that plasticizers reduce the thermal and mechanical properties of PLA, especially if the content is high [4]. Therefore, the aim of this study was to find a plasticizer content that can minimize the negative effect on the mechanical properties but still enhances the dispersion and distribution of ChNC and, consequently, obtain PLA/ChNC nanocomposites with improved properties. For this purpose, PLA nanocomposites with 3 wt % ChNC and varied triethyl citrate (TEC) contents (2.5, 5.0, and 7.5 wt %) were prepared using liquid-assisted extrusion. The control samples of PLA with similar TEC contents were prepared for the comparison. The effect of the TEC content on the ChNC dispersion and, thus, on the nanocomposite structure and properties were studied. Furthermore, the effect on the PLA molecular weight of water, solvent, and use for the ChNC feeding was also analyzed. This study shows that plasticizer plays an important role as a dispersive aid in the processing of PLA nanocomposites via liquid-assisted extrusion and that the plasticizer content should be at least 7.5 wt % to achieve well-dispersed and distributed nanocrystals. 

## 2. Experimental Section

### 2.1. Materials

Polylactic acid (PLA) (Ingeo 4043D grade) from NatureWorks LLC (Minnetonka, MN, USA) in pellet form was used as the matrix. Chitin powder from yellow lobster shell waste, purified at Pontifical Catholic University of Chile following the process reported in our earlier study [17], was used as the starting material for isolation of chitin nanocrystals (ChNC). These nanocrystals were used as to reinforce the PLA with and without the addition of a plasticizer. Liquid triethyl citrate (TEC) with a *M*w of 276.3 g/mol (≥99% Alfa Aesar GmbH & Co KG, Karlsruhe, Germany) and ethanol (99.5%) was purchased from Solveco (Stockholm, Sweden). TEC was used to enhance the ChNC dispersion in the PLA matrix, and ethanol was used as a solvent for TEC, since it is partially soluble in water and to control the flowability of the suspensions for the liquid feeding. In addition, plasticizer and ethanol were the liquid media for feeding ChNC into the extruder. 

### 2.2. Preparation of Chitin Nanocrystals and Suspensions for Liquid Feeding

Chitin nanocrystals (ChNC) were isolated via the acid-hydrolysis treatment according to the procedure reported earlier by Salaberria et al. [14]. Briefly, the chitin flakes were hydrolyzed with 3 M HCl Panreac (Barcelona, Spain) at 100 ± 5 °C under stirring for 90 min. After hydrolysis, the suspension was diluted with distilled water, washed via centrifugation and transferred to dialysis membranes for 3 days. Finally, the suspension was subjected to ultrasonic treatment for 10 min to disintegrate the remaining larger particles and then vacuum filtered using a polyamide filter Sartorious Biolab Products (Göttingen, Germany) with a 0.2 µm pore size to obtain a ChNC gel with a solid content of 19.5 wt %. 

**Figure 1:**
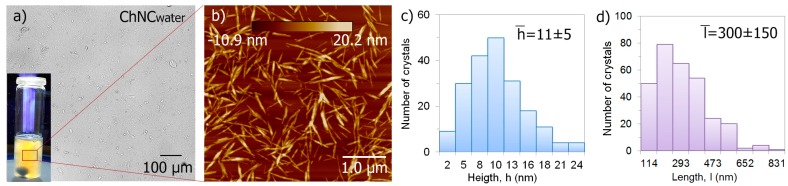
ChNC characteristics: (**a**) an optical microscopy image and a photograph displaying flow birefringence of the aqueous ChNC suspension; (**b**) a height AFM image showing the shape of ChNC; (**c**,**d**) diameter (height) and length distributions indicating the average width (h) and average length (l).

Figure 1a shows an optical microscopy image of well-dispersed ChNC in water and a photograph of chitin nanocrystals displaying flow birefringence due to good dispersion. The AFM image in Figure 1b displays the typical rod-shaped ChNC with diameters in the range of 2–24 nm, which are shown as height distribution in Figure 1c, and with lengths in the range of 114–831 nm, which are shown as length distribution in Figure 1d. The width and length were measured using the Nanoscope V software Veeco (Santa Barbara, CA, USA) and the “FibreApp” (Zurich, Switzerland) respectively. 

To feed the nanocrystals in liquid form, suspensions containing ChNC in water, TEC plasticizer and ethanol were prepared as follows: ChNC gel in water (19.5 wt %) was pre-dispersed in ethanol at a ratio of 1:5 water to ethanol for 2 h using magnetic stirring, and then mixed with TEC for 2 h. The same amount of the ChCN gel was added to all suspensions to prepare nanocomposites with a 3 wt % of ChNC, and the TEC content was varied in each suspension such that the final amount of plasticizer in the nanocomposites would be 2.5, 5.0, and 7.5 wt %. A suspension without a plasticizer was prepared for the extrusion of the unplasticized nanocomposite. Each suspension was ultrasonicated UP400S, Hielscher (Teltow, Germany) for 2 min in an ice bath prior to the extrusion and then pumped into the extruder. Mixtures of water, ethanol and TEC with the same proportions were prepared for the extrusion of plasticized PLA materials (control samples), as well as a mixture of only water and ethanol for the extrusion of PLA (control sample for unplasticized composite). 

The prepared nanocomposites are coded as PLA-TEC (the number indicates the amount of plasticizer)-ChNC, the unplasticized composite is named as PLA-ChNC, and PLA always makes reference to extruded PLA under the presence of water and ethanol, and it will be indicated otherwise. 

### 2.3. Extrusion of Nanocomposites

**Figure 2:**
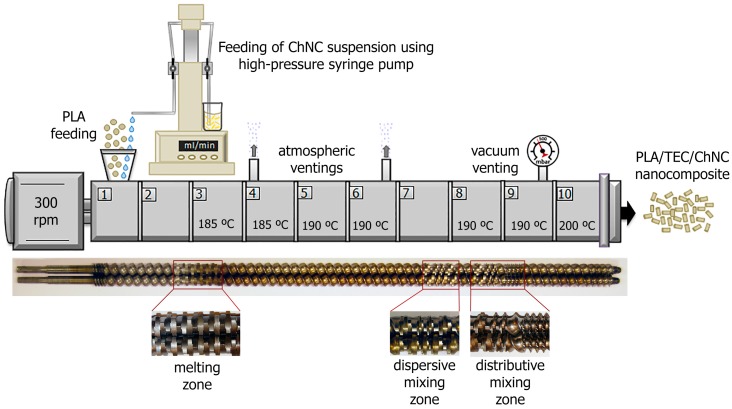
Schematic representation of the liquid-assisted extrusion process where a high-pressure syringe pump was used to feed the chitin nanocrystals suspensions into the main feeding section and the photographs of the screw design showing the melt, dispersive, and distributed mixing zones.

PLA, plasticized PLA materials (PLA-TEC), unplasticized nanocomposite (PLA-ChNC) and plasticized nanocomposites (PLA-TEC-ChNC) were prepared using a co-rotating twin-screw extruder ZSK-18 MEGALab, Coperion W&P (Stuttgart, Germany) with a liquid-assisted feeding of suspensions with a slight modification of the process described by Herrera and co-workers [9]. A K-tron gravimetric feeder (Niederlenz, Switzerland) was used to feed PLA, and a high-pressure syringe pump 500D, Teledyne Isco (Lincoln, NE, USA) was used for the liquid feeding of suspensions with ChNC and solutions without ChNC. A schematic representation of the process with the parameters and the screw configuration used are shown in Figure 2. The total throughput of the process was 2 kg/h, the screw speed was set to 300 rpm, and the temperature profile was ranging from 185 to 200 °C. The PLA pellets and suspensions were fed at the main feeding zone with a specific feeding rate for each particular material according to the final composition, as shown in Table 1. 

Two atmospheric venting and vacuum venting along the extruder were used to remove water and ethanol, as well as the trapped air. The extruded materials were cooled down in a water bath and then pelletized and dried at 55 °C overnight. The pelletized materials were compression molded using a hot press LPC-300 Fontijne Grotnes (Vlaardingen, Netherlands) to prepare films of approximately 200 µm thickness for further characterization. The pellets were placed inside metal plates covered with Mylar^®^ films Lohmann Technologies Ltd (Milton Keynes, UK) and compression molded at 190 °C for 210 s at contact pressure and then for 30 s at 4 MPa. The films were immediately removed from the metal plates and air-cooled to room temperature (~2–5 min) to avoid crystallization.

### 2.4. Characterization

#### 2.4.1. Weight

The effect of water, ethanol, TEC plasticizer and ChNC on the molecular weight of PLA was evaluated via gel permeation chromatography (GPC) using an Ultimate 3000 HPLC system (Thermo Scientific, Germering, Germany). The columns use are as follows: four Phenogel GPC columns, from Phenomenex, with a 5 µm particle size and 1E5, 1E3, 100, and 50 Å porosities, respectively. Tetrahydrofuran at a flow rate of 1 mL/min was chosen as the mobile phase, and mono-disperse polystyrene standards were used for the universal calibration.

#### 2.4.2. Melt Flow

The melt flow index of the prepared materials was measured using a melt indexer MI-1 Göttfert (Buchen, Germany). The measurements of the pelletized compounds were performed at least three times at 190 °C with a 2.16 kg load, and the average value in grams per 10 min is reported.

#### 2.4.3. Transparency

Light transmittance of the materials was measured using a Perkin Elmer UV/VIS Spectrometer Lambda 2S (Überlingen, Germany). The scan was carried out in duplicated from 200 to 800 nm with a scan speed of 240 nm/min.

#### 2.4.4. Dispersion and Morphology

The overview of the dispersion and distribution of ChNC in liquid feeding suspensions, as well as nanocomposite films were studied using a Nikon Eclipse LV100NPOL polarizing optical microscope (Shanghai, China). In the case of the nanocomposite films, cryogenic fracture surfaces were also analyzed using a FEI Magellan 400 XHR-SEM (Hillsboro, OR, USA). A thin layer (~10 nm) of tungsten was sputter-coated on the surfaces to avoid charging.

#### 2.4.5. Chemical Characterization

Fourier transform infrared spectroscopy (FT-IR) studies were performed to determine the interaction between the PLA matrix and chitin nanocrystals and the effect of further addition of TEC. The samples were ground and mixed with KBr to prepare pellets. The spectra were collected using a VERTEX 80, Bruker Corp. (Billerica, MA, USA) with a resolution of 4 cm^−1^ with 128 scans in the range of 400–4000 cm^−1^.

#### 2.4.6. Thermal Properties and Crystallinity

The thermal properties of materials were measured using a differential scanning calorimeter DSC 821e, Mettler Toledo (Schwerzenbach, Switzerland). Approximately 3 mg of the material was heated in a semi-hermetic pan from −20 to 200 °C. The tests were performed with a heating rate of 10 °C/min under nitrogen atmosphere. The degree of crystallinity (*X*c) of the films was calculated following the equation [28]:

*X*c = (Δ*H*m − Δ*H*cc)/Δ*H*m^0^ × (100/*w*) (1)

where Δ*H*m is the enthalpy of melting (pre-melt crystallization was subtracted from the melting enthalpy), Δ*H*cc is the enthalpy of cold crystallization, Δ*H*m^0^ is the enthalpy of melting for a 100% crystalline PLA sample, which is assumed to be 93 J/g [29], and *w* is the weight fraction of PLA in the sample.

#### 2.4.7. Thermo-Mechanical Properties

The thermo-mechanical properties of prepared materials were determined using a TA Instruments Q800 DMA (New Castle, DE, USA) on the 5 mm × 30 mm specimens. The experiments were performed in tensile mode from 25 to 100 °C with a heating rate of 1 °C/min and a constant frequency of 1 Hz. The testing was performed in duplicates.

#### 2.4.8. Mechanical Testing

The tensile properties of prepared materials were measured using a Shimadzu AG-X universal tensile testing machine (Kyoto, Japan) with a 1 kN load cell. The 5 mm × 80 mm specimens were cut using a rectangular press mold and then conditioned for 24 h at room conditions (25 ± 2 °C and 25% ± 2% of relative humidity). The gauge length was 20 mm, and the crosshead speed was 2 mm/min. The values for stress and elongation at break were directly obtained from the testing results, and modulus of each sample and the work of fracture were calculated from the stress-strain curves. Moreover, the properties of extruded PLA without water and ethanol were also measured and reported to analyze the effect of water and ethanol on the mechanical properties of neat PLA. The average value of five tests was reported. One-way analysis of variance (ANOVA) followed by the Tukey-HSD multiple comparison tests with a 5% significance level was used to analyze the results.

## 3. Results and Discussion

### 3.1. Suspensions for Liquid Feeding

**Figure 3:**
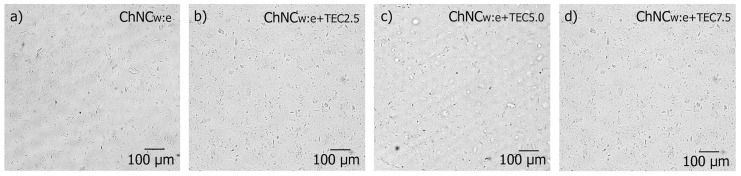
Optical images of suspensions for liquid feeding showing the dispersion of ChNC in (**a**) water and ethanol (w:e) and in (**b**–**d**) water, ethanol, and TEC plasticizer at varied concentrations.

Prior to the extrusion, the dispersion of the ChNC in the prepared suspensions was studied using an optical microscope and compared to the aqueous ChNC suspension (Figure 1a) to see the effect of ethanol and TEC. Figure 3 shows that ChNC dispersed in water, ethanol, and TEC at different concentrations are similar compared with the aqueous ChNC dispersion shown in Figure 1a. 

This confirms that the addition of ethanol and TEC did not significantly affect the dispersion of ChNC in the suspensions. All ChNC suspensions showed good stability before the extrusion. However, it is worth noting that the viscosity of suspensions was affected by the addition of the plasticizer. Suspension with the highest TEC content (7.5 wt %) resulted in the highest viscosity. The possible reason can be the better dispersion of ChNC, which was not evident at the optical microscope scale, or more interactions between TEC and ChNC.

### 3.2. Molecular Weight

The influence of water, ethanol, TEC, and ChNC, as well as of all of them together on the molecular weight of PLA was studied using GPC, and the average molecular weights *(M*_w_*)* are shown in Table 2. When comparing *M*_w_ of unprocessed PLA (as received) with *M*_w_ of extruded PLA with and without water and ethanol, it is observed that the extrusion process affects the molecular weight of PLA more than the feeding of water and ethanol. This can be attributed to a decrease in local shear due to the plasticizer effect of water [6]. *M*_w_ of the extruded PLA with water and ethanol was similar to that of the unprocessed PLA pellets (*M*_w_ ~ 199 kg/mol), showing that water and ethanol did not degrade PLA even if it is known that PLA is susceptible to hydrolytic degradation. When PLA was extruded with water, ethanol and TEC, the presence of TEC increased the molecular mobility of PLA, which may increase the water diffusion rate into the PLA molecules and thus, enhances the hydrolytic degradation [30], which results in a PLA-TEC5.0 material with a somewhat lower molecular weight (*M*_w_ ~ 196 kg/mol) but still less degraded that the extruded PLA. 

When comparing the molecular weight in Table 2 of unplasticized composite (PLA-ChNC) and plasticized nanocomposite (PLA-TEC5.0-ChNC), the values show that the reduction in molecular weight of PLA due to the addition of ChNC in a water ethanol suspension (from 199 to 181 kg/mol) was more than that due to the addition of ChNC in water, ethanol and TEC suspension (from 199 to 193 kg/mol). It is possible that, in general, the presence of additives, such as ChNC, may increase the thermo-mechanical degradation of PLA due to higher shear forces, as has been reported by others [6,31]. This effect may be smaller in a presence of a plasticizer. It is concluded that in this study, the polymer degradation due to chitin was hindered by the use of plasticizer and that the addition of chitin promoted the polymer degradation more than the addition of water and ethanol. Similarly, Stoclet et al. [6] reported that the processing of PLA/halloysite nanocomposites via conventional extrusion (dry method) resulted in higher degradation of PLA than the water assisted extrusion process, where the injection of water decreased the effect of the halloysite on the PLA molecular weight. In contrast, Rizvi et al. [32] reported hydrolytic degradation of PLA when it was processed with chitin in water suspension in a micro-compounder. However, the difference between that study compared with the present one is the long processing time, and the micro-compounder does not effectively remove the water and/or solvents, and the authors did not use a plasticizer. The processing time in Rizvi’s study was 6 min, while the resident time in this study is less than 1 min, which may not be enough time to promote the hydrolysis of PLA. It should also be noted that the extrusion process involving liquids works better as a continuous process than as a batch process and with extruders with an appropriate degassing system.

### 3.3. Melt Flow 

The measurement of the melt flow index (MFI) of the prepared materials gives indirect information about the dispersion and interaction between the polymer and nanocrystals since the flow behavior of polymer nanocomposites is influenced by the interfacial characteristics and the nanoscale structure [33]. The effect of the addition of varied amounts of TEC on the flow properties of PLA and PLA-ChNC was evaluated and the MFI values are listed in Table 2. The results show that the plasticized PLA exhibited higher MFI than PLA, as expected. The MFI of PLA was 3.1 g/10 min, and PLA-TEC7.5 showed the highest value of 4.9 g/10 min due to the highest amount of the plasticizer. The addition of TEC increases the polymer free volume and the polymer chains’ mobility and, thus, decreases the viscosity and increases the MFI which is a typical effect of the plasticizer [34]. 

Opposite to the effect of the plasticizer, the addition of nanocrystals restricts the polymer chains’ mobility and, thus, the MFI of the matrix decreases. It is seen from Table 2 that all nanocomposites, except for the PLA-ChNC, exhibited lower MFI than their respective materials without ChNC. The addition of ChNC to the PLA-TEC7.5 material decreased its MFI from 4.9 to 3.7 g/10 min, showing the largest effect. This result is an indication that the dispersion and interaction of the nanocrystals in the PLA-TEC7.5-ChNC nanocomposite were better than the nanocomposites with lower TEC contents. On the other hand, the PLA-ChNC composite showed a higher MFI than PLA, which indicates that the interaction of nanocrystals with the matrix was poor. In this case, the higher MFI can also be due to the lower molecular weight of the PLA-ChNC composite.

### 3.4. Transparency and Visual Appearance

**Figure 4:**
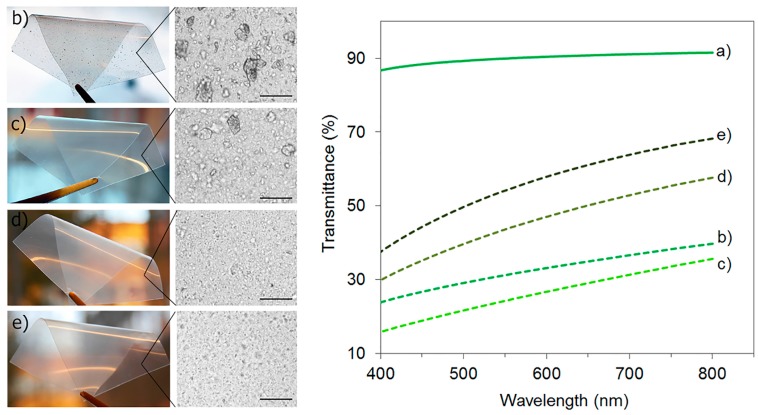
To the left, the photographs of nanocomposite films with their optical microscopy images (scale bar 100 µm), and to the right, UV/VIS spectra of: (**a**) extruded PLA with water and ethanol; (**b**) PLA-ChNC; (**c**) PLA-TEC2.5-ChNC; (**d**) PLA-TEC5.0-ChNC; and (**e**) PLA-TEC7.5-ChNC.

The visual appearance of the extruded PLA with water and ethanol and its nanocomposite films as well as the optical microscopy images of the film surfaces are shown in Figure 4 (to the left). It is clear that the unplasticized composite shows visible agglomeration, which is not observed in plasticized nanocomposites. However, the optical microscopy images also show micro-sized agglomerations for the PLA-TEC2.5-ChNC nanocomposites but not for the nanocomposites with 5.0 wt % and 7.5 wt % TEC. The optical transparency of materials was measured because it can give an indication of the dispersion and distribution of ChNC in PLA. It is known that if the size of particles is smaller than the wavelength of visible light, the transparency of the matrix is affected less [35]. It was noticed during the test that the addition of TEC did not affect the PLA transparency, and the light transmittance spectra were overlapping with that of PLA. Therefore, those UV/VIS spectra are not displayed in Figure 4 (to the right), but the spectra of the extruded PLA with water and ethanol and its nanocomposites are shown. It is observed that the light transmittance of PLA decreased with the addition of chitin nanocrystals. At 550 nm of visible light, the light transmittance of PLA was 90%, whereas it was only 52%, 44%, 24%, and 30% for the PLA-TEC7.5-ChNC, PLA-TEC5.0-ChNC, PLA-TEC2.5-ChNC, and PLA-ChNC materials, respectively. These results show that the nanocomposites with the highest TEC content (7.5 wt %) had the best transparency of the nanocomposites and, thus, expected to have the best dispersion of ChNC which is in accordance with the MFI results. 

### 3.5. Morphology of Nanocomposites and ChNC Dispersion 

**Figure 5:**
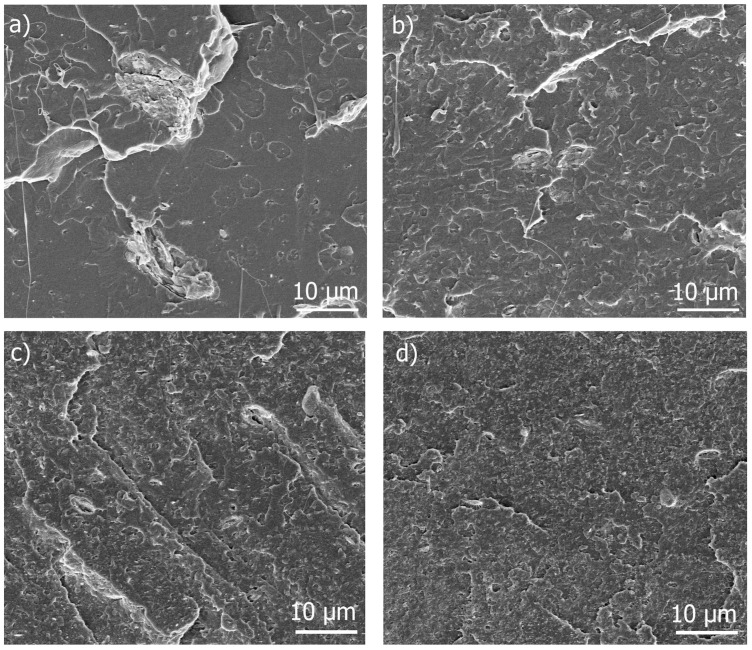
HR-SEM images of the cryogenic fracture of: (**a**) PLA-ChNC; (**b**) PLA-TEC2.5-ChNC; (**c**) PLA-TEC5.0-ChNC; and (**d**) PLA-TEC7.5-ChNC showing improved dispersion and distribution of ChNC with an increased TEC content.

Figure 5 displays the cryogenic fracture surface of the unplasticized PLA-ChNC composite and the nanocomposites with a different TEC content. These micrographs clearly show that the dispersion and distribution of ChNC gradually improved with the plasticizer content as was also seen in the transparency and MFI studies. 

**Figure 6:**
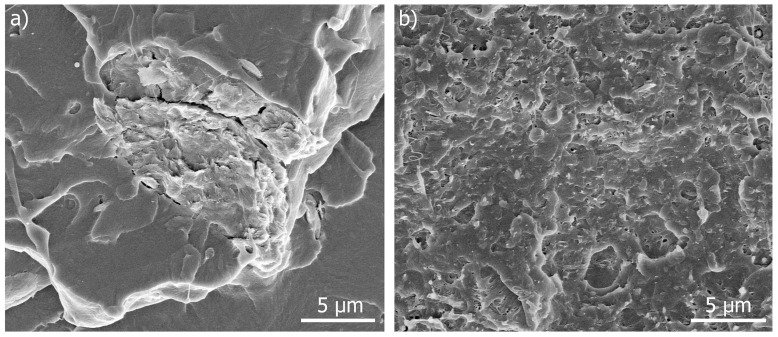
HR-SEM images at higher magnification of (**a**) PLA-ChNC showing poor dispersion and distribution of ChNC and (**b**) PLA-TEC7.5-ChNC showing better dispersed and distributed ChNC as white dots.

The micrograph at higher magnification for the PLA-ChNC composite (Figure 6a) shows poor dispersion and distribution and large agglomeration (~10 µm) of ChNC, whereas the PLA-TEC7.5-ChNC nanocomposite, with the highest TEC content (Figure 6b), exhibits more even, well-dispersed and distributed chitin nanocrystals with few agglomerations which are much smaller than those in Figure 6a. These results are in agreement with our previous studies, where the addition of poly(ethylene glycol) (PEG) enhanced the dispersion of cellulose nanocrystals [23] and with the results reported by Wang et al. [24] and Qu et al. [25] who have reported that acetyl tributyl citrate (ATBC) and PEG enhanced the dispersion of carbon black and cellulose nanofibers in PLA, respectively.

### 3.6. Chemical Charaterization

**Figure 7:**
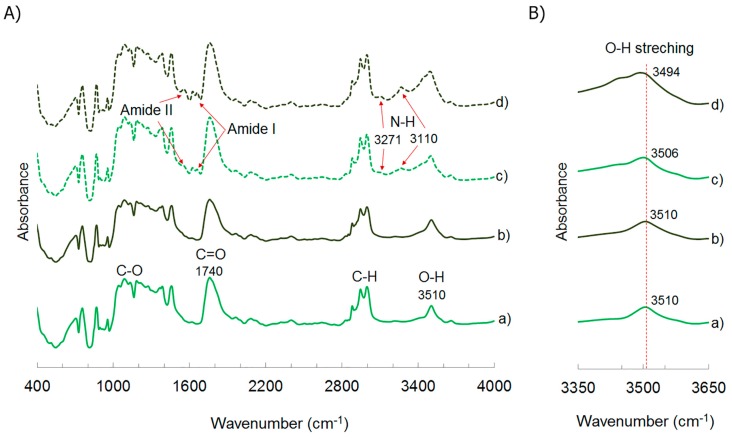
(**A**) FTIR spectra of: (a) extruded PLA with water and ethanol; (b) PLA-TEC7.5; (c) PLA-ChNC and (d) PLA-TEC7.5-ChNC showing the PLA and chitin characteristic peaks and (**B**) zoomed view of the FTIR peak of O-H stretching showing the shift of the O-H peak to a lower wavenumber when ChNC or ChNC together with TEC were added to PLA.

The effect of addition of TEC in the interaction between PLA and ChNC was analyzed using FTIR. Figure 7A shows infrared spectra of extruded PLA with water and ethanol, PLA-TEC7.5, PLA-ChNC, and PLA-TEC7.5-ChNC. 

The characteristic peaks of PLA were observed in all analyzed materials. The peak at 1760 is attributed to the carbonyl (–C=O) stretching of PLA. The peaks between 2850 and 3000 cm^−1^ belong to the C–H asymmetric and symmetric stretching vibration [36]. The peak of the –C–O– bond stretching in –CH–O– and in –O–C=O of PLA appear at 1182 and 1081 cm^−1^, respectively [24]. The peaks at 1621 and 1656 cm^−1^ and at 1556 cm^−1^ correspond to the amide I and II [37], respectively. The peaks at 3110 and 3271 cm^−1^ are ascribed to the N–H stretching [38]. The above mentioned data confirmed the presence of chitin in the composites. From the PLA spectra, a peak at approximately 3510 cm^−1^ can be seen, which is related to the O–H bond stretching deformation. This indicates the presence of hydroxyl groups in pure PLA [39]. This peak did not change with the addition of TEC. However, this peak was broader and slightly shifted to a lower wavenumber (3506 cm^−1^) when ChNC were added to PLA, and it further broadened and shifted to 3494 cm^−1^ when ChNC was added together with TEC, as can be seen in Figure 7B. These results indicate the H-bonding interactions between PLA and ChNC. Rosdi and Zakaria [40] also found that the peak at 3505 cm^−1^ was shifted to a lower wavenumber when chitin was added to the PLA matrix, possibly due to some interaction between the hydroxyl groups of PLA and the hydroxyl groups of chitin. The results also indicate that the H-bonding interactions between PLA and ChNC were enhanced in the presence of TEC. It is believed that TEC may help the intermolecular interaction between PLA and chitin and enhances their interfacial interaction, which is in agreement with the SEM images. Similar results have been reported by Qu et al. [25], who showed that PEG improved the intermolecular interaction between PLA, PEG, and cellulose. No new peaks were detected when TEC or ChNC were added to PLA or when TEC was added to the PLA-ChNC nanocomposite.

### 3.7. Thermal Properties and Crystallinity

**Figure 8:**
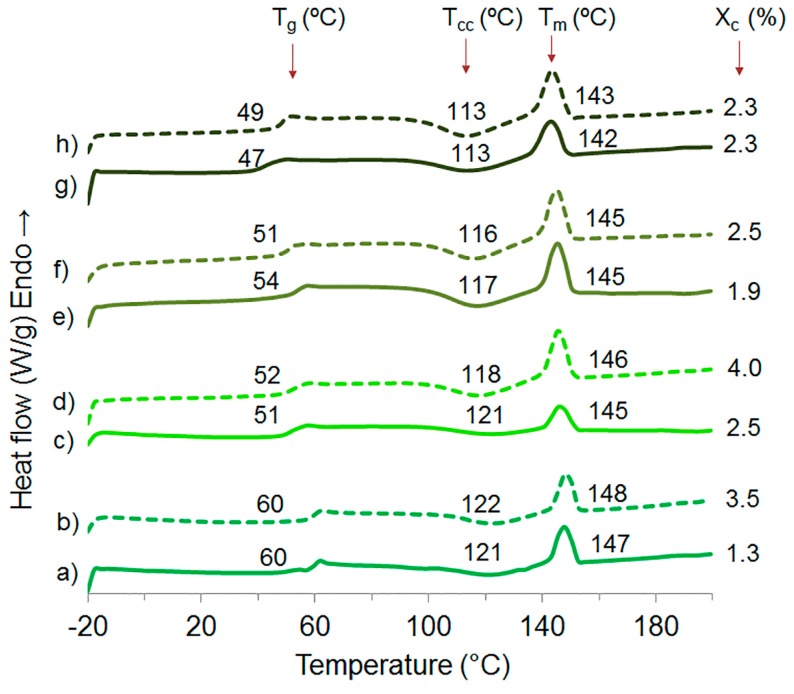
DSC scans of: (a) extruded PLA with water and ethanol; (b) PLA-ChNC; (c) PLA-TEC2.5; (d) PLA-TEC2.5-ChNC; (e) PLA-TEC5.0; (f) PLA-TEC5.0-ChNC; (g) PLA-TEC7.5; and (h) PLA-TEC7.5-ChNC.

DSC thermograms and glass transition (*T*_g_), cold crystallization (*T*_cc_) and melt (*T*_m_) temperatures of extruded PLA with water and ethanol, plasticized PLA materials and nanocomposites are shown in Figure 8. All presented *T*_g_, *T*_cc_, and *T*_m_ for the materials indicate their semi-crystalline nature. The *T*_g_, *T*_cc_, and *T*_m_ of PLA are 60, 121 and 147 °C, respectively, and these values decreased with 7.5 wt % TEC content to 47, 113, and 142 °C. The decrease is because of the plasticizing effect [41]. *T*_g_, *T*_cc_, and *T*_m_ of the plasticized PLA materials remained almost the same with the addition of ChNC, and only a slight increase of *T*_g_ from 47 to 49 °C was observed for the material with a 7.5 wt % of TEC. This slight improvement of the glass transition temperature may be due to a better interaction between PLA, TEC and ChNC in this nanocomposite, which hinders the polymer molecular mobility. Figure 8 shows the degree of crystallinity (*X*_c_) where the addition of TEC and ChNC did not show any significant effect. 

### 3.8. Thermo-Mechanical Properties

**Figure 9:**
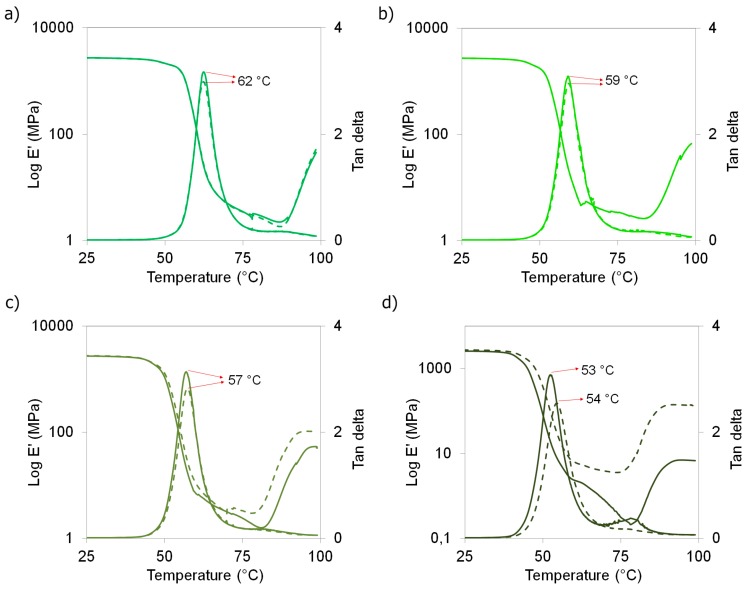
Storage modulus (left Y-axis) and tan delta peaks (right Y-axis) as a function of temperature of PLA nanocomposites (dashed lines) and their respective counterpart materials without nanocrystals (continuous lines). (**a**) unplasticized materials; (**b**) plasticized materials with 2.5 wt % TEC; (**c**) plasticized materials with 5.0 wt % TEC; and (**d**) plasticized materials with 7.5 wt % TEC.

Figure 9 shows storage modulus and tan delta (δ) as a function of temperature for PLA nanocomposites and their counter-parts without nanocrystals with different TEC contents as well as those for the unplasticized materials. In Figure 9a, PLA and PLA-ChNC are compared. It is observed that the addition of ChNC did not affect the storage modulus or tan delta peak position. Respectively, in Figure 9b–d, the plasticized nanocomposites with 2.5, 5.0 and 7.5 wt % TEC content are compared with their respective counterpart without ChNC. Similar to the DSC results, only the PLA-TEC7.5-ChNC nanocomposite showed a slight increase in the tan δ position. In addition, a decrease in the intensity of the peak was also observed. A positive shift in tan δ commonly indicates restricted molecule movement, and a decreased intensity of tan δ shows that lower number of polymer chains participates in the transition, which is expected because of the well-dispersed and distributed nanocrystals in the PLA-TEC7.5-ChNC nanocomposite. This better ChNC dispersion is also reflected in an improved storage modulus. These results indicate that the nanocomposites with the highest TEC content (7.5 wt %) shows better dispersed and distributed nanocrystals and, thus, slightly enhanced thermo-mechanical properties.

When comparing PLA with the plasticized PLA materials, it is observed that the increased TEC content in PLA decreases the tan delta peak position towards lower temperature from 62 °C to 53 °C with the addition of 7.5 wt % TEC, which confirms the plasticizer effect of TEC. Moreover, it is seen that the increased TEC content together with ChNC enhances cold crystallization, and higher TEC content is more effective that the lower TEC content. 

### 3.9. Mechanical Properties

The mechanical properties of PLA nanocomposites and their counter-part materials without nanocrystals are reported in Table 3. In addition, the mechanical properties of the extruded PLA without water and ethanol are reported, and if comparing these properties with those from the extruded PLA in the presence of water and ethanol, no significant effect on the mechanical properties of PLA was noticed. 

It is possible to see in Table 3 that the addition of TEC decreased the tensile strength of PLA and did not increase the elongation at break or work of fracture, as expected. These results indicate that a higher amount of plasticizer is required to obtain a noticeable effect on the toughness. Labrecque et al. [4] reported that all citrate esters are effective in improving the elongation at break at higher concentrations (≥20%), but do not show any significant increase at lower concentration. However, both DSC and DMA results showed that the plasticizer contents used in this study were enough to plasticize PLA. 

The nanocomposites with TEC ≥ 5 wt % showed higher tensile strength and ultimate strength than the respective plasticized PLA without ChNC. Moreover, these materials showed slightly improved Young’s modulus based on the ANOVA test. The elongation at break and work of fracture were decreased in all cases except for the nanocomposite with 7.5 wt % of TEC. This is due to less agglomeration and better dispersion of ChNC in the PLA-TEC7.5-ChNC nanocomposite. 

The decrease observed in the tensile strength of PLA-ChNC can be attributed to the hydrolysis of PLA during the processing [32], which was observed in the GPC results, and because of the presence of micro agglomerations with a poor interface, as observed in the SEM studies. These results are similar to the results reported by Hishammuddin and Zakaria [36] where the incorporation by mixing, and then casting of commercial chitin into PLA, resulted in reduced tensile strength and elongation. Salaberria et al. [20] also reported a slight decrease of mechanical properties of PLA when functionalized (acylation) chitin nanocrystals were introduced into PLA via extrusion/compression. Rizvi et al. [32] found that the stiffness of PLA increased with increasing chitin content while the strength was found to decrease. However, in this study, it was found that the addition of ChNC into PLA together with TEC showed enhanced mechanical properties when ≥5.0 wt % of plasticizer was used. This is explained because the dispersion and distribution of ChNC and their interaction with the PLA matrix was improved with increasing plasticizer content as it has been shown in the previous sections of this paper. Similarly, Li et al. [18] reported that PEG worked as a compatibilizer for chitin nanofibers and PLA when it was used as pretreatment for the nanofibers before the compounding process.

## 4. Conclusions

This study was carried out to determine a plasticizer content that has the minimum plasticizer effect on PLA, but still enhances the dispersion and distribution of ChNC in the PLA matrix and, thus, obtain a nanocomposite with improved properties. Therefore, PLA composites with 3 wt % of chitin nanocrystals and triethyl citrate with varied contents of 2.5, 5.0, and 7.5 wt % were produced via liquid-assisted extrusion.

The gel permeation chromatography confirmed that the addition of water and ethanol during the extrusion process did not significantly affect the molecular weight of PLA. 

The liquid feeding of ChNC together with TEC plasticizer resulted in PLA-TEC-ChNC nanocomposites with improved dispersion and distribution of ChNC. The nanocomposite with the highest plasticizer content (PLA-TEC7.5-ChNC) showed enhanced mechanical, thermal, and thermo-mechanical properties, compared with its counter-part without ChNC (PLA-TEC7.5). The improved interaction between PLA and ChNC in the presence of TEC is attributed to hydrogen bonding, which was supported by the FTIR study. 

It will be interesting to study the effect of a higher plasticizer content to determine the synergic effect of the plasticizer as a dispersing and toughening aid with a minimum impact on the properties of PLA. The presented facile process of nanocomposites using liquid-assisted extrusion with a plasticizer, which facilities nanomaterial dispersion, can be a step forward for a large-scale production of bionanocomposites. 

## Figures and Tables

**Table 1:** Processing conditions for the liquid-assisted extrusion process showing the final material compositions.

Materials	Feeding Rate (kg/h)		Composition of Materials (wt %)		
	PLA	Suspension	PLA	TEC	ChNC
PLA	2.00	1.56 ^1^	100	-	-
PLA-ChNC	1.94	1.56 ^2^	97.0	-	3
PLA-TEC2.5	1.95	1.55 ^3^	97.5	2.5	-
PLA-TEC2.5-ChNC	1.89	1.61 ^4^	94.5	2.5	3
PLA-TEC5.0	1.90	1.60 ^3^	95.0	5.0	-
PLA-TEC5.0-ChNC	1.84	1.66 ^4^	92.0	5.0	3
PLA-TEC7.5	1.85	1.65 ^3^	92.5	7.5	-
PLA-TEC7.5-ChNC	1.79	1.71 ^4^	89.5	7.5	3

^1,2,3,4^ Fed into extruder, and 1.50 kg/h of water and ethanol were removed as vapor during extrusion. ^1^ Water and ethanol; ^2^ Water, ethanol, and ChNC; ^3^ Water, ethanol, and TEC; ^4^ Water, ethanol, TEC, and ChNC.

**Table 2:** Molecular weight (*M*w) and melt flow index.

Materials	Molecular Weight (kg/mol)	Melt Flow Index (g/10 min)
PLA ^1^	199	-
PLA ^2^	186	-
PLA ^3^	199	3.1
PLA-ChNC	181	3.5
PLA-TEC2.5	-	3.2
PLA-TEC2.5-ChNC	-	2.7
PLA-TEC5.0	196	4.3
PLA-TEC5.0-ChNC	193	3.8
PLA-TEC7.5	-	4.9
PLA-TEC7.5-ChNC	-	3.7

^1^ Unprocessed PLA, as received in pellets; ^2^ Extruded PLA; ^3^ Extruded PLA in the presence of water and ethanol.

**Table 3:** Mechanical properties of PLA nanocomposites and their counter-part materials without chitin nanocrystals.

Materials	Young’s Modulus (GPa)	Tensile Strength (MPa)	Ultimate Strength (MPa)	Elongation at Break (%)	Work of Fracture (MJ/m^3^)
PLA ^1^	1.86 ± 0.10	60.6 ± 0.6	52.0 ± 4.8	7.9 ± 0.4	3.5 ± 0.3
PLA ^2^	1.87 ^a^ ± 0.05	59.2 ^a^ ± 2.1	55.2 ^a^ ± 3.3	5.8 ^a^ ± 0.4	2.3 ^a^ ± 0.2
PLA-ChNC	1.91 ^a^ ± 0.08	52.7 ^b^ ± 0.7	50.2 ^a^ ± 1.7	4.5 ^b^ ± 0.3	1.6 ^b^ ± 0.2
PLA-TEC2.5	2.04 ^α^ ± 0.13	54.7 ^α^ ± 3.9	51.6 ^α^ ± 3.9	5.6 ^α^ ± 0.6	2.2 ^α^ ± 0.3
PLA-TEC2.5-ChNC	1.90 ^α^ ± 0.03	56.6 ^α^ ± 0.9	55.3 ^α^ ± 1.3	4.2 ^β^ ± 0.3	1.4 ^β^ ± 0.2
PLA-TEC5.0	1.80 ^δ^ ± 0.04	49.4 ^δ^ ± 2.5	44.7 ^δ^ ± 2.0	6.5 ^δ^ ± 0.8	2.4 ^δ^ ± 0.3
PLA-TEC5.0-ChNC	1.94 ^γ^ ± 0.02	54.4 ^γ^ ± 1.7	52.3 ^γ^ ± 1.8	4.6 ^γ^ ± 0.1	1.6 ^γ^ ± 0.1
PLA-TEC7.5	1.83 ^A^ ± 0.00	43.7 ^A^ ± 0.6	39.8 ^A^ ± 3.5	5.6 ^A^ ± 0.9	1.8 ^A^ ± 0.4
PLA-TEC7.5-ChNC	1.92 ^B^ ± 0.02	47.5 ^B^ ± 0.2	45.8 ^B^ ± 0.1	5.3 ^A^ ± 0.5	1.9 ^A^ ± 0.3

^1^ Extruded PLA; ^2^ Extruded PLA with water and ethanol. Same superscript letters (a, b, α, β, δ, γ, A and B) within the same column are not significantly different at 5% significance level based on ANOVA and the Tukey-HSD multiple comparison test.
